# Development and evaluation of a COVID tracking system to support provision of social service in Wyandotte County, Kansas

**DOI:** 10.3389/fpubh.2023.1035319

**Published:** 2023-06-23

**Authors:** Catherine Wexler, Katelyn Sanner Dixon, Kevin Oyowe, Brooke Lapke, Hannah Conner, Hailey Shoemaker, Erin Corriveau, Allen Greiner, Sarah Finocchario-Kessler

**Affiliations:** ^1^Department of Family Medicine, University of Kansas Medical Center, Kansas City, KS, United States; ^2^School of Medicine, University of Kansas Medical Center, Kansas City, KS, United States; ^3^Global Health Innovations–Kenya, Nairobi, Kenya; ^4^Unified Government of Wyandotte County Health Department, Kansas City, KS, United States

**Keywords:** COVID-19, eHealth, RE-AIM, human centered design, qualitative research

## Abstract

**Background:**

In addition to the state-mandated case investigation and contact tracing, the Unified Government Public Health Department of Wyandotte County, Kansas implemented social support services for COVID-19 cases and contacts; however, did not have the systems in place to document the provision of these services. Our team worked with the health department to develop and implement the COVID Tracking System (CTS), an eHealth system that linked multiple involved teams. Here, we describe the development and evaluation of the CTS. The objective of this manuscript is to describe and evaluate the development and implementation process of the Covid Tracking System.

**Methods:**

Drawing from concepts of user-centered design, we took a 4-phase approach to development: understanding context, specifying needs, designing solutions, and evaluating. A mixed-methods evaluation of the development and implementation process using RE-AIM was conducted. Quantitative CTS data captured between February 1, 2021, and September 30, 2021 were exported. Descriptive statistics were calculated for categorical variables and means (SD, range) or median (IQR) for continuous variables. Qualitative discussions with key users supplemented the quantitative data.

**Results:**

There were 1,152 cases entered into the CTS, of whom 307 (26.6%) requested a letter be sent to their workplace to excuse them during their quarantine period, 817 (70.9%) requested and had food and cleaning supplies delivered, 21 (1.8%) requested guidance on applying for federal assistance, and 496 (43.1%) requested to be contacted by a community health worker. While a few technical glitches slowed down early implementation, these were quickly resolved and key users felt that the CTS streamlined client referral and simplified their workflow, allowing them to spend more time on patient care and follow up, rather than documentation. After study implementation ended, the Unified Government Public Health Department of Wyandotte County continued using the CTS for client tracing and follow up.

**Discussion:**

This project provides a roadmap of how user centered design can be applied to the development and evaluation of eHealth software to support program intervention implementation, even in situations where urgent action is needed.

## 1. Introduction

The first confirmed case of COVID-19 appeared in Kansas in Johnson County on March 7, 2020 and in Wyandotte County on March 12, 2020 (1). The number of cases grew rapidly, with 326,000 confirmed cases in the state and 27,068 cases in Wyandotte County as of September 30, 2021 (1). The state of Kansas mandated the use of a system called EpiTrax—a system that was already in place for any reporting of communicable diseases (TB, HIV, etc.)—for statewide COVID-19 reporting, case management, and contact tracing. Recognizing that social stability and health inequity impacts health outcomes and ability to adhere to medical recommendations, the Unified Government of Wyandotte County, Kansas (UGPHD) put in place systems to reach beyond the basic public health response of tracking and monitoring disease spread. UGPHD began implementing social support services for COVID-19 cases and contacts (2). Additional support offered to those positive for COVID-19 and their contacts included: linkage to primary care and/or mental health services, delivery of food and cleaning supplies during their quarantine period, and the opportunity to speak with a community health worker (CHW) for further assistance with social needs.

**Figure 1:**
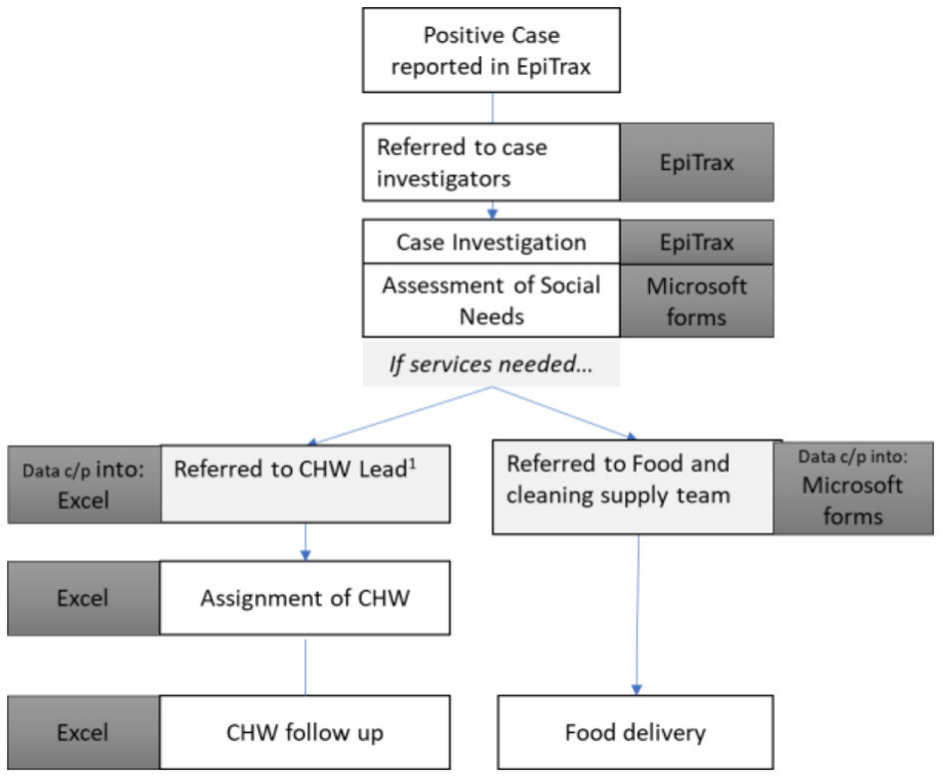
Flow of Covid-19 clients through Wyandotte county social services, pre-CTS. Each team within the health department had their own customized—but unlinked—form, creating duplication of effort and the opportunity for error.

Each patient with COVID-19 received a thorough assessment of social needs. First, case investigators conducting the initial case investigation and contact tracing assessed clients' needs. Referrals to the CHW team and/or food and cleaning supply team were then made accordingly. EpiTrax did not have the flexibility to track these unique referrals, so the local health departments developed tools including Microsoft Forms and Excel sheets to track the social needs of their resident clients. The tools used by each team (case investigators, CHWs, food/cleaning supply team) were unlinked and referrals from one team to another required the copying and pasting of information, which was time consuming, created duplicate client entries in each source, and was prone to error. Figure 1 outlines the flow of clients through social services, as well as the unlinked tracking tools utilized by Wyandotte County prior to the implementation of the Covid Tracking System (CTS).

Drawing from concepts of user-centered design, (3) our team worked with the UGPHD to develop the COVID Tracking System (CTS)—a single system that would fully meet the varying needs of each of the three teams' involvement in providing enhanced social support to clients. Guided by the RE-AIM model, (4) the objectives of this manuscript are to describe the development and implementation process of the CTS to support programmatic implementation of these social services and assess facilitators and barriers to CTS use among key stakeholders. Such studies outlining the rapid deployment of technology to enable program implementation and data collection during the pandemic are rare. These data demonstrating how robust systems can be developed based on unique user needs will be valuable to program implementers and system developers.

## 2. Methods

### 2.1. Study overview

This study was part of an intervention development study to design and pilot the COVID Tracking System to meet user needs in Wyandotte County, Kansas. The CTS was collaboratively developed by members of the UGPHD, academic partners, and Global Health Innovations, which provided the technical programming and system maintenance. CTS implementation began January 2021 and continued through 2022.

### 2.2. User centered design

User centered design is a product development framework, which prioritizes the user and their needs at each phase of product design and development. In this four-phased development process, developers: (1) understand context of use, (2) specify user requirements, (3) design solutions, and (4) evaluate against requirements (3, 5). Conversations around developing the system began in July 2020. Guided by principles of user centered design, our development and implementation process took a four-phase approach:

#### 2.2.1. Phase 1. Understanding the continuum of COVID-19 follow-up in Wyandotte County

Starting in August of 2020, developers held routine meetings with key stakeholders in UGPHD, including epidemiologists, lead case investigators, lead CHW, and health officers. It was originally envisioned as a tool to assist laboratory tracking of results, case investigation and follow up, and contact tracing. However, it became clear in early conversations that the state mandated EpiTrax served that function and could not be replaced. Further, a pathway to link or import data to EpiTrax was unclear. Thus, the focus of the CTS shifted during these initial conversations to build a system both urgently but also within the context of an already existing data collection system.

### 2.3. Phase 2. **Specifying user requirements**

After the context of implementation was understood, developers worked with stakeholders to define their needs. Meetings were held with each team that participated in the COVID-19 social needs response to understand their current tools, required data, preferred formatting, and considerations for client tracking, reporting, automated client linkage to other teams, and additional features that could streamline their workflow (i.e., automated letter generation). Additional requirements in terms of data storage, user login types, and suggested reporting formats were specified and developed later in the process.

#### 2.3.1. Phase 3. Designing solutions

The design and development phase occurred concurrently with phase 2, with the technical programmer frequently meeting with Wyandotte County team members to show progress on the system, solicit feedback and recommendations for improvement, and nuance specifications for user requirements.

The final product—the CTS—was a system designed to link the workflows of the three teams (case investigators, CHW, and food and cleaning suppliers), allowing relevant information to be automatically routed from one team to another for follow up. Within the system, there was a unique dashboard for each team. These dashboards only showed client data that was relevant to the user but were linked to each other through a unique client ID—allowing information relevant to all users to be transferred from one dashboard to the other. Case investigators entered data simultaneously into EpiTrax and CTS while conducting the initial case investigation. Upon initial contact with the client, case investigators used a check box to indicate if CHW follow up was requested and/or if food and cleaning supplies were needed. Clients who needed these services were then automatically added to the respective team's dashboard for follow up. In total, the CTS replaced 7 distinct Excel sheets and Microsoft forms, with 1 system. Additional features of the CTS included “alerts” when follow-up was needed (e.g. unfilled food and cleaning supply requests, unfulfilled CHW follow up, federal assistance requests, etc.), automatic generation of workplace follow up letters, and easy data export for reporting and data analysis.

**Figure 2:**
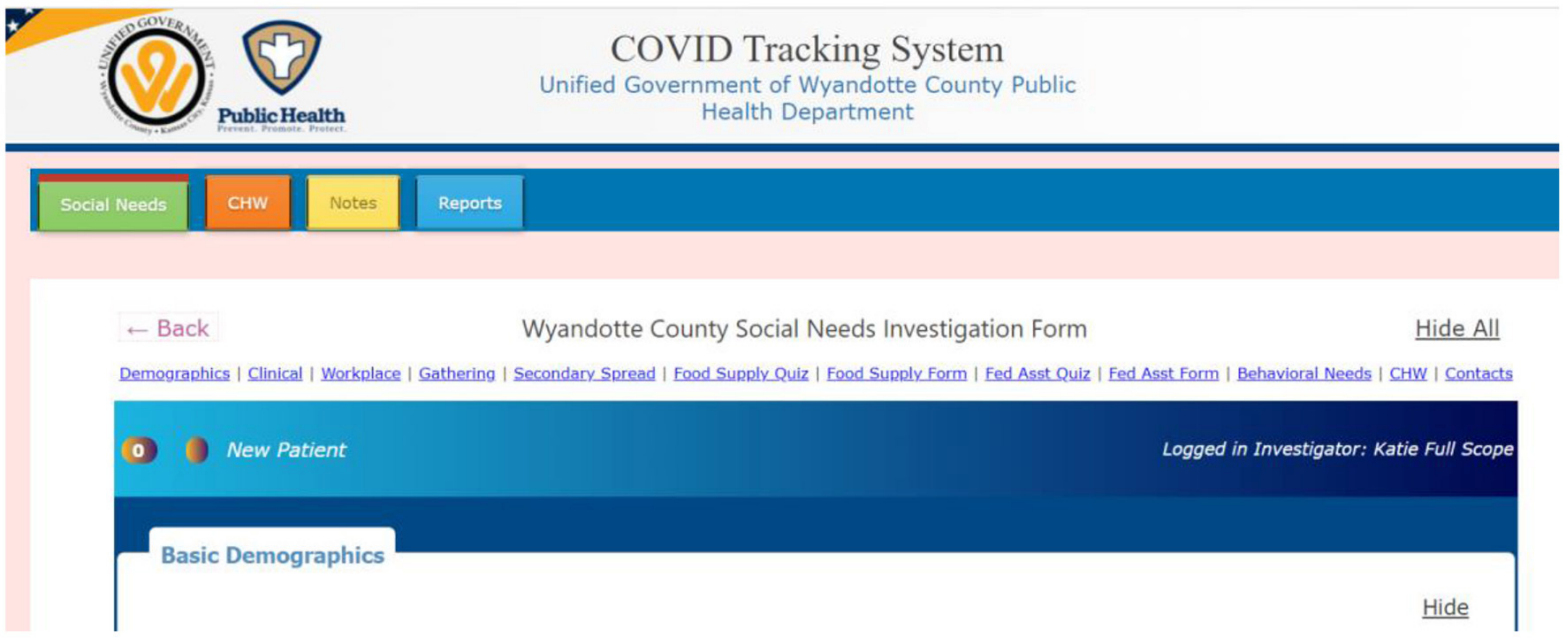
CTS user dashboard. Social needs tracking, community health worker tracking, food and cleaning supply reports were streamlined to a single tool. The notes tab allowed teams to easily communicate with each other within the system.

The CTS was piloted during a 1-week trial by key users prior to implementation to ensure full functionality and make final updates to ensure integration with workflow. During this 1-week period, training materials (including a training video and a user manual) were developed and circulated to the team. Figure 2 displays the user dashboard of the final CTS.

#### 2.3.2. Phase 4. Evaluating the intervention

An evaluation of the design and implementation process was conducted after approximately three months of CTS use and is described in this manuscript.

### 2.4. Evaluation model and measurements

The RE-AIM model provides a framework for evaluating the implementation of interventions in real-world settings, using five key dimensions: reach, effectiveness, adoption, implementation, and maintenance (4). Each of the components of RE-AIM are defined in Table 1, and a brief overview of the measurements used in our study are described. Methods specific to each measure type are described below.

**Table 1:** RE-AIM construct definitions and their measurements within this study.

	**Definition**	**Measurement**
Reach	The absolute number, proportion, and representativeness of individuals who are willing to participate in a given initiative, intervention, or program	Quantitative CTS data
Effectiveness	Impact of an intervention on important outcomes, including potential negative effects, quality of life, and economic outcomes	Qualitative interview data
Adoption	The absolute number, proportion, and representativeness of settings and intervention agents (people who deliver the program) who are willing to initiate a program	Qualitative interview data Quantitative CTS data
Implementation	Fidelity to the various elements of an intervention's protocol, including consistency of delivery as intended and the time and cost of the intervention	Field notes Qualitative interview data
Maintenance	The extent to which a program or policy becomes institutionalized or part of the routine organizational practices and policies	Field notes Qualitative interview data

### 2.5. Quantitative CTS data

Quantitative data captured by the CTS between February 1, 2021, and September 30, 2021 were exported to Excel using the system's automated reporting functions. Descriptive statistics were calculated for categorical variables and means (SD, range) or median (IQR) for continuous variables.

### 2.6. Qualitative interviews

Key personnel of the UGPHD who were part of the COVID-19 response and/or who were familiar with the CTS were eligible to be interviewed. Between May 10 and May 28, 2021, we conducted 3 key informant interviews and 3 focus group discussions with key stakeholders at UGPHD. Key informant interviews were held with the two lead epidemiologists and the lead community health worker. The three focus group discussions (FGD) were held with (1) case investigators, (2) community health workers, and (3) the food and cleaning supply team. In total, we talked with 12 participants. No eligible participant refused to participate or dropped out. Table 2 displays information about the composition of interviews and focus groups, as well as roles of participating individuals.

**Table 2:** Overview of key informant interview and focus group discussion characteristics.

	**Type**	**Participant(s)**	**Participant(s) role**	**Number of participants**	**Length**
1	Interview	Epidemiologist 1	Data reporting. Contacting businesses regarding potential outbreaks.	1	8:10
2	Interview	Epidemiologist 2	Managing case investigators	1	26:13
3	Interview	Lead community health worker	Assign clients to community health workers for follow up. Also serves as a community health worker	1	20:59
4	FGD	Case investigators	First point of contact with clients. Conduct initial case investigation and data entry. Link clients to CHWs and food and cleaning supplies	4	42:24
5	FGD	Community health workers	Follow up with clients requesting services to assist with linkage to care, financial assistance, etc.	3	38:51
6	FGD	Food and cleaning supply team	Organize deliveries of food and cleaning supplies to client who request it.	2	26:43

To maintain social distancing during the ongoing pandemic, all communication with study participants were virtual. A co-investigator (KSD) reached out to potential participants with information about the study via email and asked if they would be willing to participate. If they agreed, a follow up email was sent with a study information sheet and with the timing of the interview and/or focus group discussions. Given the minimal risk and virtual nature of this study, the Institutional Review Board at University of Kansas Medical Center waived the need for written informed consent. All participants provided verbal consent for participation, including audio recording of the interview.

Interviews took approximately 10–30 min and focus group discussions took 30–45 min. The qualitative guide was developed for this study and questions included topics such as: participants role in the COVID-19 response, challenges they've experienced in that role, ways to mitigate challenges, how the CTS was used in their role, impact of the CTS on workflow and patient follow up, challenges with the CTS, and suggested modifications to the CTS, see Supplementary material 1. All interviews and focus group discussions were conducted by a Co-Investigator (KSD, female, medical student and lead case investigator), who helped form the COVID-19 response and co-managed Covid-19 case investigators and, thus, was familiar with study participants. No repeat interviews were conducted. All coding took place in Microsoft Excel. Participants did not provide additional feedback on the findings.

#### 2.6.1. Qualitative analysis

All interviews and focus groups took place and were recorded via Zoom and then transcribed verbatim. We used a content analysis approach to identify and interpret key themes from participant responses. An initial codebook was developed based on a priori themes related to CTS use. Transcripts were coded and memo-ed independently by 4 analysts (KSD, BL, SFK, CW) using Excel. Transcript coding was an iterative process with cross-questioning and critique between the coders. A final codebook included themes that emerged with exemplars for each theme and the frequency and distribution of themes within the larger topic areas noted.

## 3. Results

We present the results of CTS reach, effectiveness, implementation, adoption/maintenance to evaluate the initial impact of the CTS for social needs support in the context of COVID-19.

### 3.1. Reach

**Figure 3:**
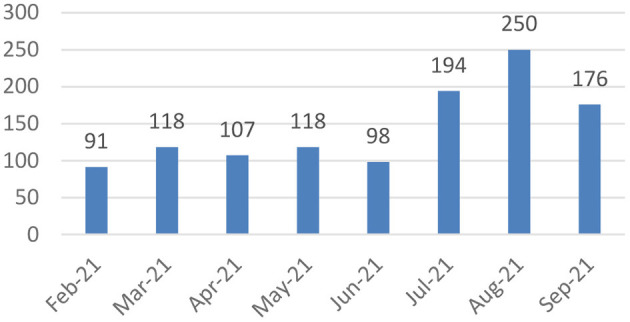
CTS enrollment by month.

Between February 1, 2021, and September 30, 2021, there were 1,152 cases entered into the COVID Tracking System (Figure 3). Of these 728 were female (63.2%) and 424 were male (36.8%). The median age of clients was 30.4 years old (IQR: 17.8–44.3). Clients came from 14 unique zip codes across the Kansas City Metropolitan area.

Of the 1,152 cases, the CTS automatically generated 307 letters be sent to clients' workplaces to excuse them during their quarantine period, documented delivery of 817 food and cleaning supplies, helped linked 21 clients to guidance on applying for federal assistance, and linked 496 clients to the CHW dashboard.

The CTS documented community health worker outreach to the 495 of the 496 clients who requested follow up, and describes a total of 1,118 outreach encounters recorded across all participants. Each participant received 0–13 follow up calls from a CHW, with a median of 1 encounter per client. Of the 496 who requested CHW follow up, 345 have complete follow up documented, while 38 later declined CHW follow up, 73 clients are still in active CHW follow up through the CTS, 39 were unable to be reached, and 1 has not yet been contacted. Issues discussed between clients and CHWs included food and product assistance, income and/or utility assistance, health insurance, mental health, and housing. Each of CHW to client call is documented within the CTS, as is the status of CHW follow up, allowing CHWs to more easily track and manage their client load.

### 3.2. Effectiveness

#### 3.2.1. CTS benefits

Effectiveness of the CTS to streamline user workflow was assessed qualitatively. Overall, users felt the CTS was an effective way to track the social services provided to clients. Prior to CTS, there was no single method or system in place to house all the information for contact tracing, including an assessment of clients' social needs. Rather, contact tracers used Microsoft Forms, while community health workers used Excel sheets. CTS allowed for a transition to one system for record keeping, creating a more efficient process. Users described the benefit of having client information in one place, rather than spread out over different sheets. Additionally, its automatic transfer of client information between teams reduced duplication of data entry.

*In the past, I was receiving the Excel forms from the contact tracers and I had to sort it and clean it up to be able to pull just the information that I needed it to create the referrals for the team, and then I had to divide by language to assign to [CHW1] or [CHW2] or myself and then I had to copy and paste in at least three different Excel forms, and then when I was assigning the clients myself, then I have to put the information on the call log who I was talking to and also if anybody requested food and cleaning supplies, I had to copy and paste all that information into another Excel forms. So I don't know, it was maybe seven Excel forms that I don't have to do anymore because of CTS*. (CHW3)

Case investigators and CHW described how instead of duplicating answers across unconnected forms, CTS allowed users to see client information in one place. This allowed them more time to focus on client care, rather than documentation.

“*But yes, it was just how incredible it simplifies our work, because that will give us more time to work with the clients. Now the fact that everything is organized the way that we wanted, that was, like, easy.”*

Users also mentioned that CTS was helpful in providing quarterly reporting and customization of reporting information.

*So, having the ability to just pull the report out I think has smoothed things along...”*

#### 3.2.2. CTS barriers

Despite its benefits, there were barriers to CTS use that hindered effectiveness. These barriers were mainly related to technical problems, increased workload, and need for enhanced training.

Technical problems were found to be the most common barriers to CTS use. Many of these technical problems were related to data entry (e.g., couldn't use apostrophes, required a specific browser) and issues with saving data (e.g., the system was slow at saving notes, missing or incomplete entries).

A few participants mentioned an increased workload due to the higher volume of information that needed to be entered in the CTS compared to the old system.

“*I think at first, I struggled with it. It just felt like it was just more documentation…I think it got better as it progressed. But I will still say that, still, I think there should be a way to shorten it down for us to make it a little bit faster.”*

The need for enhanced training was also noted. This prevented the users from doing things simply because they did not know how.

“*The CHWs, they say, linked to mental health, they fill out the form that is there and is wonderful, because also very convenient. But they said, “Okay, how do I send it from here?”*

Other barriers included inaccurate or outdated information, password challenges, incomplete entries in the system, redundant information, and no official contact resources page.

### 3.3. Implementation

The system was implemented on February 1, 2021, and a small group of key users (*n* = 3) piloted it for 1 week and provided feedback, prior to full scale roll out.

While the transition to CTS was challenging in the beginning, since they were familiar with the old system, users were able to adjust quickly to the CTS.

“*I think part of it also is my experience with [Microsoft] Forms because I've been working with [Microsoft] Forms for years and years. So, I just felt comfortable with the format and layout. And this was just something new. And at that point, I just felt like it was something else that was being added. So maybe, that's what it was. And that was my hesitation at first and my struggle with it. But like I said, I mean, they get easier eventually.”*

Users suggested key modification to streamline implementation. Most suggestions for improvements were minimal, with few complex requests. Requested modifications fell into two main categories: adding features and modifying features, as seen in Table 3.

**Table 3:** Summary of key user recommendations.

**Requested features to add**	**Requested features to modify**
• Providing CHWs with a count of clients they are working with	• Ability to de-select after selecting options
• Ability to upload documents like patient bills into the system	• Making workplace data and exposure tracking a dropdown
• Color coding client records based on where they are in follow up	• Free text boxes in case a patient has been linked to multiple services
• Adding fields or field options for more comprehensive data collection	• Reversing the order so more recent patients at top
• Adding alerts to remind CHWs to call a patient on a certain day	• Removing completed patients from table on dashboard

### 3.4. Adoption and maintenance

The CTS was adopted as the Health Department's social needs tracking component in February 2021 and replaced previously implemented tools. In total, 22 case investigators, 4 community health workers, 2 members of a community-based organization who delivered food and cleaning supplies, and 3 epidemiologists within the health department access the system.

As the intervention development grant that funded the development and early implementation of the CTS closes, UGPHD plans to continue using the CTS for social needs tracking. Costs associated with implementation are low, approximately $500/month, which includes retainer for the technical developer to maintain and modify the system and server space for secure data storage.

## 4. Discussion

The leadership of UGPHD noted that social barriers were impacting their clients' ability to adhere to COVID-19 public health and medical recommendations. Thus, as part of their public health response, UGPHD began offering social services, including food and cleaning supply delivery, linkage to CHWs who could offer guidance on health insurance, and referrals for income and utility assistance to clients. UGPHD needed a more streamlined solution to document these services. Working with academic and non-profit partners, the Covid Tracking System was developed to urgently address the need for a data entry system that met the needs of three separate teams working to provide social services. From February 2021 through September 2021, more 1,000 clients and the services they received were documented in the CTS. Furthermore, the system was highly acceptable to key users and long-term implementation was maintained after study completion.

User-centered designed has been applied to COVID-19 applications both domestically and internationally. These applications have curated content for COVID-19 vaccine campaigns, (6) enhancing communication with the public, (7) displaying critical health information to clinical providers, (8) and supporting task shifting from primary facilities to community health workers (7). Like our study, the iterative process of development and implementation for these interventions focused on understanding the context of implementation and user needs. Additionally, it allows stakeholders to provide frequent feedback during development and early implementation. In our study, this granted us the ability to quickly shift away from our initially envisioned scope (assisting laboratory tracking of results, case investigation and follow up, and contact tracing) which was already documented in the state-mandated system to a scope that focused on the current needs of the health system: streamlining their documentation on the social-needs of their clients. This led to a system that was widely accepted by key users, and ultimately adopted and maintained by end users. This highlights how the application of user-centered design can the product to ensure the product meets the needs of the end user, while also being feasible to implement in the context it was designed for.

This manuscript highlights the user-centered design process of CTS development to support the provision of social need services in the context of the COVID-19 pandemic. However, it is not without limitations to generalizability. In the absence of pre-intervention or control data, quantitative data can only be analyzed descriptively. Without comparative data, we do not know whether this descriptive data reflects increased needs and resolution of these needs because of the intervention. We used qualitative interviews to describe the processes in place and allow stakeholders to reflect upon the pre-intervention period; however, cannot support this with quantitative data. All interviewees were associated with UGPHD, either as employees or volunteers, and there was no way to anonymize interviews. Thus, there may have been some degree of social desirability bias reflected in our results. Furthermore, due to the extremely overburdened staff and funding limitations, most local health departments in Kansas were not attempting to systematically address social needs during the acute phases of the COVID-19 pandemic. Thus, the success of the system itself may be difficult, but certainly not impossible, to generalize beyond use in Wyandotte County, KS, since it was their unique services that drove system development. Aspects of the CTS could be customized to support a range of feasible services in various settings with the capacity to link staff at other community organizations and ultimately build and strengthen community partnerships in emergency response.

## 5. Conclusion

The application of concepts from user-centered design allowed for the rapid development and implementation a system that filled noted gaps within the provision of COVID-19 social services in Wyandotte County, Kansas. Using an iterative development process and a simultaneous evaluation of implementation using components of the RE-AIM model helped ensure that the system fit the context for which it was implemented, even when that context changed during the evolving pandemic. With increased recognition of the role social factors play on individual and population health outcomes, the design and development of systems to document the provision of these services is necessary to securely organize, manage and analyze a large amount of data. The process of design and implementation of the CTS could provide a needed resource for health departments, community clinics, or hospitals who are looking to develop systems to comprehensively document their programs. This project provides an example of how user centered design can be applied to eHealth, even in situations where urgent action is needed.

## Data availability statement

The raw data supporting the conclusions of this article will be made available by the authors, without undue reservation.

## Ethics statement

The studies involving human participants were reviewed and approved by University of Kansas Medical Center Institutional Review Board. Written informed consent for participation was not required for this study in accordance with the national legislation and the institutional requirements.

## Author contributions

CW managed study implementation, contributed to qualitative and quantitative analyses, and drafted the manuscript. KSD conceived of the study, conducted qualitative interviews, and managed programmatic implementation. KO managed intervention design, development, and maintenance. BL conducted qualitative analysis and assisted with drafting of the manuscript. HC, HS, EC, and AG provided input on system design and oversaw programmatic implementation. SF-K conceived of the study and oversaw all study implementation. All authors provided input on the draft manuscript and approved of the final manuscript.

## Supplementary material

Supplementary Material 1 CORE-Q checklist for qualitative research.
